# A New Substance

**Published:** 1886-12

**Authors:** 


A NEW SUBSTANCE.—A new use has been discovered for potatoes. They can be converted into a substance resembling celluloid by peeling them, and after soaking in water, impregnating with eight parts of sulphuric acid, then drying and pressing between sheets of blotting paper. In certain parts of France pipes are made of this substance scarcely distinguishable from meerschaum. By subjecting the mass to great pressure billiard balls can be made of it rivalling ivory in hardness.

